# Therapeutic Response to PRRNT in a Rare Case of Metastatic Renal Neuroendocrine Carcinoma

**DOI:** 10.1055/s-0044-1785461

**Published:** 2024-04-09

**Authors:** Saumya Sara Sunny, Julie Hephzibah, Raju Titus Chacko, Thomas Alex Kodiatte

**Affiliations:** 1 Department of Nuclear Medicine, Christian Medical College and Hospital, Vellore, Tamil Nadu, India; 2 Department of Medical Oncology, Christian Medical College and Hospital, Vellore, Tamil Nadu, India; 3 Department of Pathology, Christian Medical College and Hospital, Vellore, Tamil Nadu, India

**Keywords:** neuroendocrine tumors, peptide receptor radionuclide therapy, theranostics, lutetium-177, DOTATATE

## Abstract

Neuroendocrine tumors (NETs) are a rare spectrum of neoplasms that are characterized by neuroendocrine and neural differentiation. The treatment can be challenging in view of the heterogeneity in differentiation and behavior. Primary renal origin NETs are rare and only a few cases have been reported in the literature. There is limited knowledge on their presentation and response to various lines of treatment. We report a case of a patient with a metastatic renal NET from a rare histological subtype of large cell neuroendocrine carcinoma, known to cause aggressive disease with poor prognosis. A multimodality treatment approach was followed. In spite of surgical management and second-line chemotherapy, the disease progressed. The patient subsequently received peptide receptor radionuclide therapy (PRRNT) using lutetium-177 DOTATATE, following which the patient demonstrated a remarkable clinical and radiological response and is stable to date. In a rare tumor with poor prognosis, the relevance of theranostics and the efficacy of targeted therapies like PRRNT are noteworthy.

## Case Presentation

### History and Examination

A gentleman in his early 20s presented with complaints of left flank pain for 2 months associated with fatigue and weight loss. He had no comorbidity, significant history, or family history.

On examination, a mass was palpable in the left hypochondrium measuring 5 × 6 cm. It was not tender, and had a smooth surface and firm consistency. Fingers could be insinuated between the mass and the costal margin and was bimanually palpable. The rest of the systemic examination was unremarkable.

### Investigations

Contrast-enhanced computed tomography (CT) of the abdomen showed an 8 × 10 × 10 cm lobulated mass arising from the lower pole of the left kidney and abutting the psoas muscle.

He underwent a left transperitoneal radical nephrectomy under general anesthesia. Intraoperatively, a lobulated mass (measuring 10 × 7 cm) arising from the lower pole of the left kidney was noted. Adhesions were found on the medial side of the kidney with no palpable left hilar lymphadenopathy, intraperitoneal nodules, or free fluid.

**Fig. 1:**
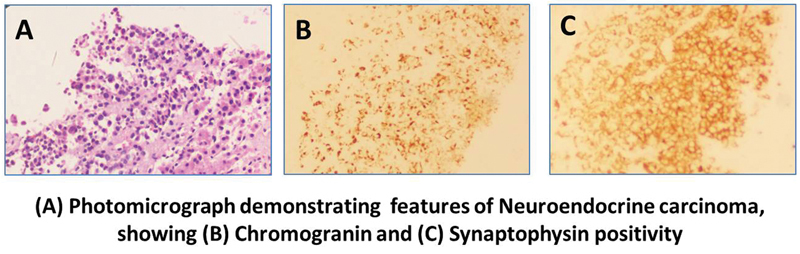
( **A** ) Photomicrograph demonstrating features of neuroendocrine carcinoma showing ( **B** ) chromogranin and ( **C** ) synaptophysin positivity.

The biopsy was reported neuroendocrine carcinoma, large cell type, in the left nephrectomy specimen. The gross specimen showed a tumor in the lower pole measuring 10 × 7 × 8 cm with a variegated cut surface. The tumor was composed of lobulations with a tan brown appearance and grayish white areas with foci of calcification, congestion, and necrosis. Microscopically, there was necrosis, atypia, and increase in mitotic activity with MIB1 index greater than 20% in the poorly differentiated areas. The immunohistochemistry profile showed positivity for vimentin, panCK, synaptophysin, and chromogranin ( Fig. 1 )

During the multidisciplinary team (MDT) meeting, it was decided that the patient's urinary catecholamines and urinary 5-hydroxyindoleacetic acid (HIAA) levels should be tested, and a ^68^ Ga-DOTATATE (gallium-68 1,4,7,10-tetraazacyclododecane-1,4,7,10-tetraacetic acid [DOTA peptide]) positron emission tomography and computed tomography (PET-CT) scan should be performed. All these were within normal limits. Following another MDT meeting, he was offered a platin-based chemotherapy in view of foci of high mitotic index, lymphovascular emboli, and MIB1 index greater than 20%. He received six cycles of adjuvant chemotherapy with cisplatin and etoposide.

His disease was stable for 2 years, after which he developed cough and abdominal pain.

**Fig. 2:**
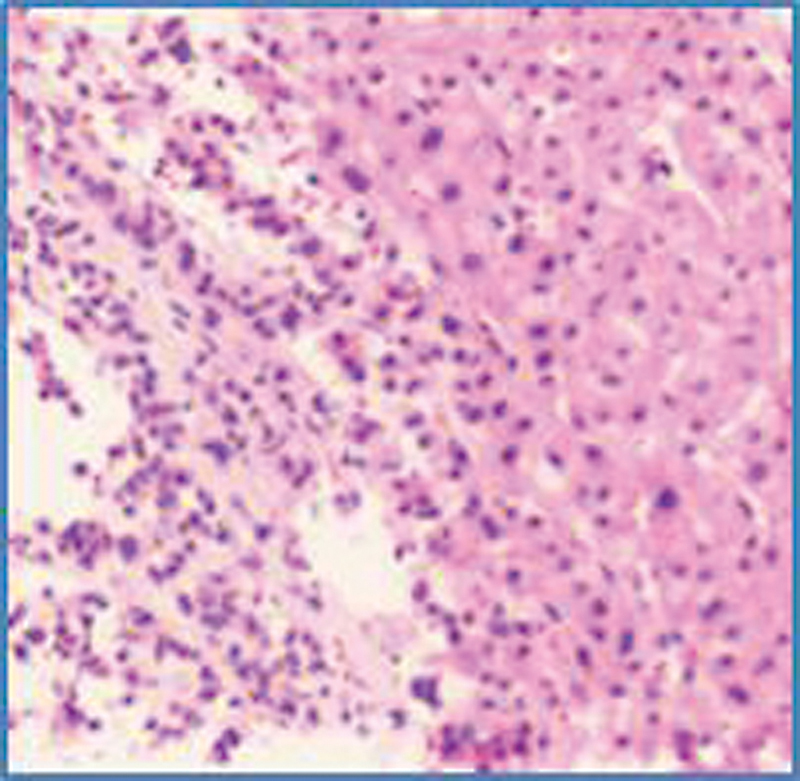
Biopsy from a new liver lesion, confirming neuroendocrine neoplasm.

CT of the abdomen done then showed multiple liver lesions and lung nodules, and a biopsy from the right lobe of the liver confirmed a metastatic neuroendocrine neoplasm ( Fig. 2 ), following which he received six cycles of second-line chemotherapy with capecitabine + temozolomide.

**Fig. 3:**
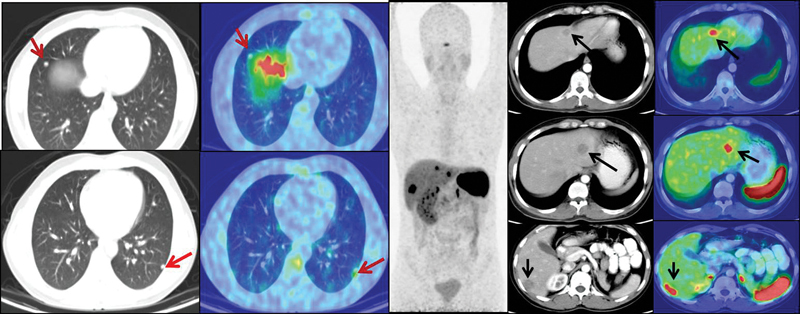
Repeat ^68^ Ga-DOTATATE positron emission tomography and computed tomography (PET-CT; axial CT, fused PET-CT, and maximum intensity projection [MIP] images) confirming avid liver lesions and nonavid lung nodules.

He underwent an iodine-131 mIBG (metaiodobenzylguanidine) diagnostic scan, but as the lesions were nonavid, I-131 mIBG therapy was deferred. A repeat ^68^ Ga-DOTATATE PET-CT showed avid liver and lung lesions with disease progression, following which he was referred to nuclear medicine for palliative intent peptide receptor radionuclide therapy (PRRNT; Fig. 3 ).

**Fig. 4:**
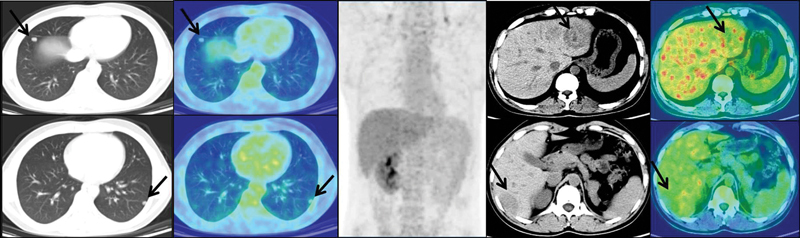
Fluorine-18 fluorodeoxyglucose ( ^18^ F-FDG) positron emission tomography and computed tomography (PET-CT; axial CT, fused PET-CT, and maximum intensity projection [MIP] images) showing nonavidity in the liver and lung lesions. The arrows denote non-DOTATATE avid lung nodules and avid liver lesions.

A fluorine-18 fluorodeoxyglucose ( ^18^ F-FDG) PET-CT was also done, but the metastatic disease was nonavid ( Fig. 4 ).

### Treatment

As ^68^ Ga-DOTATATE PET-CT imaging showed avid lesions, the patient was planned for PRRNT.

**Fig. 5:**
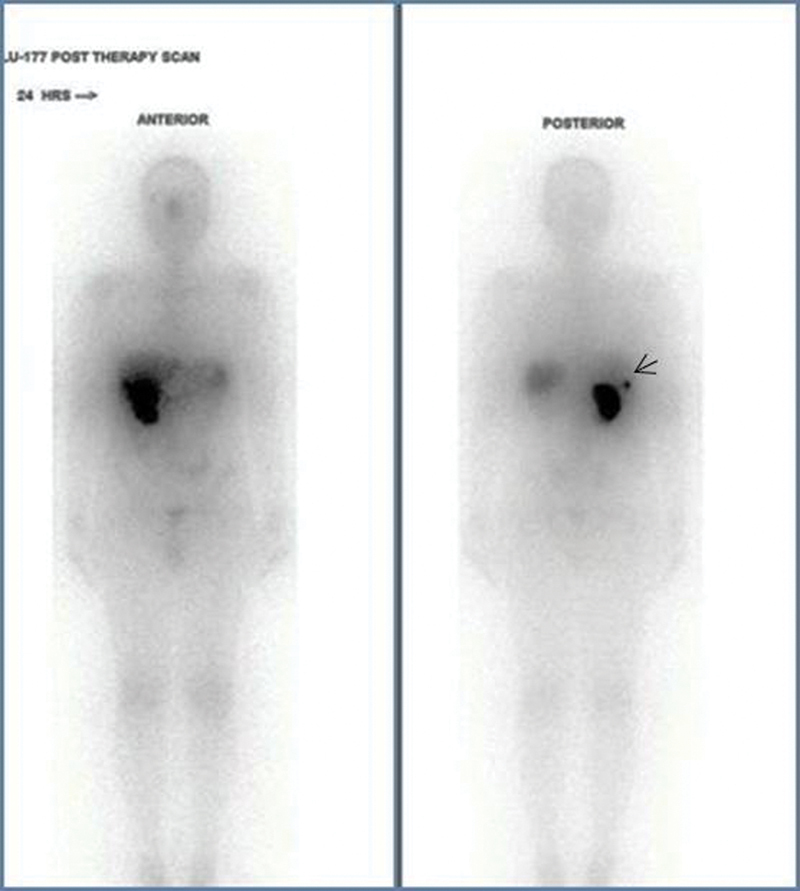
Posttherapy scan confirming uptake in the lesions of the liver.

He received four doses of lutetium-177-DOTATATE therapy, 3 months apart (February, May, August, and December 2017). During each therapy, intravenous (IV) amino acid infusion (Aminoven) was administered 30 minutes prior to the lutetium-177-DOTATATE infusion. A total of 2 L was administered over 6 hours. Lutetium-177-DOTATATE (average dose of 4,070 MBq) was diluted in 20 mL of normal saline and administered as a slow IV infusion over 20 minutes. Posttherapy imaging was done the next day after infusion to document the distribution of uptake in the avid lesions ( Fig. 5 ).

Following the third dose, repeat ^68^ Ga-DOTATATE PET-CT scans showed significant disease regression.

### Follow-Up

**Fig. 6:**
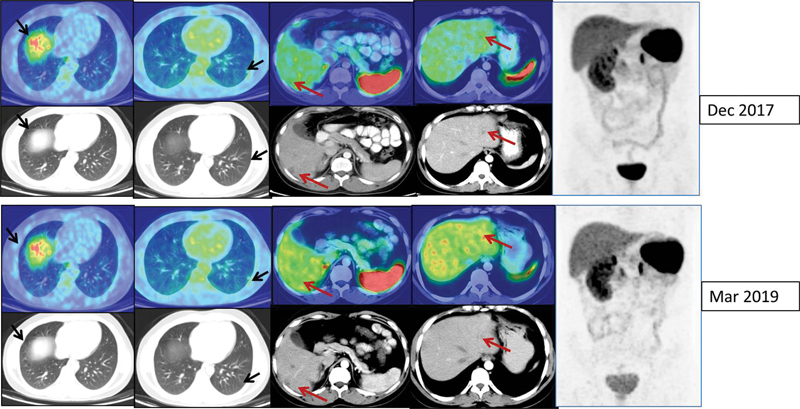
Serial ^68^ Ga-DOTATATE positron emission tomography and computed tomography (PET-CT) scans showed absence of any avid lesions with decrease in the size of the nonavid liver lesions and resolution of lung nodules. Arrows denote resolution of the previously noted lung nodules and reduction in the size and avidity of the liver lesions.

**Fig. 7:**
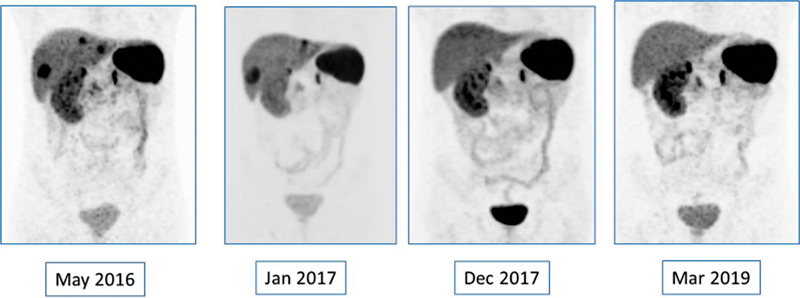
Serial maximum intensity projection (MIP) images demonstrating response.

**Fig. 8:**
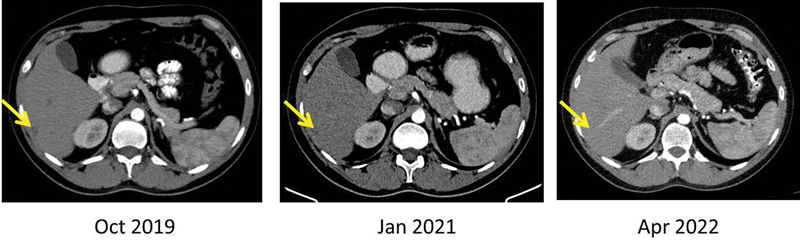
Computed tomography (CT) images were acquired in the last few visits, which showed that the liver lesion was stable. Here, Arrows show the near total resolution of the metastatic liver lesion.

Following completion of lutetium-177-DOTATATE therapy, there were no major adverse effects such as marrow suppression or renal toxicity. Subsequent follow-up visits in the next 4 years showed significant clinical improvement. Serial ^68^ Ga-DOTATATE PET-CT scans in the next 3 years showed the absence of any avid lesions with decrease in the size and uptake of liver lesions ( Figs. 6 and 7 ). Only CT images were acquired in the last few visits and the liver lesions were stable ( Fig. 8 ).

The patient showed remarkable symptomatic improvement and was able to join work and resume all normal activities as his premorbid state.

## Discussion

Neuroendocrine tumors (NETs) are relatively uncommon tumors that demonstrate variable neuroendocrine differentiation and diverse biological behavior. 1 The most common location is in the gastrointestinal tract, particularly in the stomach and small and large intestines. The occurrence in the respiratory system is less frequent. However, the origin in parenchymal organs such as the liver and primary NETs of the kidney is rare, 2 because neuroendocrine cells are not found within the normal renal parenchyma. 3

A study by Romero et al analyzed 56 case reports of primary renal carcinoid. They reported that the diagnosis was made incidentally in 28.6% of patients. The most common symptom reported was abdominal or flank pain and only 12.7% presented with neuroendocrine syndrome with symptoms of dyspnea, flushing, and diarrhea. Metastases was noted in 45.6% of patients at initial diagnosis. The peak incidence is during the fifth and sixth decades and age more than 40 years carried a poor prognosis. 4 In contrast, our patient was in the early 20s during the initial diagnosis.

Primary renal NETs, based on histopathology, are primarily of two types: well-differentiated and high-grade neuroendocrine carcinomas. 5 High-grade neuroendocrine carcinomas can be divided into large cell neuroendocrine carcinomas (LCNECs) and small cell neuroendocrine carcinomas (SCNECs), and are generally found to have poor prognosis due to the propensity for locally advanced disease and distant metastases. 6

In previous studies, due to the rarity of the tumors and the absence of carcinoid syndrome, almost 14.5% of renal NETs were initially misdiagnosed. This is particularly observed in LCNECs, due to the morphological similarities with high-grade renal cell carcinomas. Positivity for neuroendocrine markers, such as synaptophysin, chromogranin, and CD56, and the absence of CD10 positivity differentiate LCNECs from high-grade renal cell carcinomas. 7

Aggressive features on histopathology include high mitotic activity (>2/10 high power fields), K _i_ -67 index, and presence of necrosis and hemorrhage. 4 8 The patient in our case had a primary renal LCNEC (high grade) with size greater than 4 cm, high-risk features of necrosis, high MIB1 index, and high mitotic index.

Staging is done with appropriate imaging. The expression of somatostatin receptors by NET cells enables targeted uptake when injected with Ga68 DOTATATE tracers. ^18^ In addition, avid lesions, which are receptor positive on the scan, are amenable to the targeted β radiation of PRRNT to the tumor cells. 10 F18 FDG PET-CT has a complementary role in these tumors and shows avidity in poorly differentiated components of the tumor. 9

Due to the lack of randomized trials, owing to the rarity of cases, treatment decisions require an MDT approach. Nephrectomy is the treatment of choice and is often curative in low-grade renal carcinoids. A long-term close follow-up postoperatively is essential as metastasis can occur even up to 5 years after diagnosis. 11

There are no trials done to assess the direct impact on survival when nephrectomy/neoadjuvant therapies, such as chemotherapy, PRRNT, octreotide analogs, or radiotherapy, are offered. The efficacy of chemotherapy in terms of overall survival for primary renal carcinoids has not been established. 12 Octreotide analogs may be useful in functional carcinoids, and the antineoplastic property leads to improved time to progression of disease as demonstrated by the PROMID study, but this was performed in metastatic midgut NETs. 13

When PRRNT in the form of β emitters such as lutetium-177-DOTATATE therapy is administered, the radiolabeled somatostatin analogs bind to the somatostatin receptors on tumor cells. Direct targeting of tumor cells is possible by the delivery of the high energy of the β emitter, lutetium, by internalization and storage within lysosomes. 14 PRRNT, along with somatostatin analogs, has proven its efficacy in treating gastroenteropancreatic NETs, which are somatostatin receptor positive. Many studies have established improvement in symptoms and quality of life. 15 However, it has not been studied in metastatic renal NECs.

In our case, the patient underwent a platin-based chemotherapy in view of the high-risk features postnephrectomy. However, he presented with recurrence of metastatic liver lesions 2 years later. Even with the second-line chemotherapy, the disease progressed, and he was referred for PRRNT.

Less than 15 cases of LCNECs have been reported in the literature and almost all had rapid progression. 16 So far, there have been no case reports determining the treatment response of PRRNT in primary renal NETs (LCNEC subtype). We would like to highlight the favorable outcome of the treatment of a tumor associated with a dismal prognosis. This could be attributed to the targeted β radiation from lutetium-177-DOTATATE therapy to the tumor cells that express somatostatin receptors. The significant role of theranostics in changing a palliative treatment to a curative one is encouraging. Further studies must be performed to establish the efficacy of PRRNT in treating primary renal NETs and various subtypes.

## Conclusion

Renal NETs are a rare group of neoplasms that have a propensity to metastasize and carry a high risk of recurrence, especially if the mitotic index is high. Treatment decisions are generally complex and need an MDT approach. Even in disease progression, in spite of multiple lines of chemotherapy, PRRT can be offered even in aggressive tumors such as small cell carcinomas (SCCs) and LCNECs (subtype of primary renal NETs). There is always the possibility of encouraging results and clinical and symptomatic improvement with palliative PRRT, due to the targeted β radiation that specifically acts on the somatostatin receptors in NETs.
